# miRLAB: An R Based Dry Lab for Exploring miRNA-mRNA Regulatory Relationships

**DOI:** 10.1371/journal.pone.0145386

**Published:** 2015-12-30

**Authors:** Thuc Duy Le, Junpeng Zhang, Lin Liu, Huawen Liu, Jiuyong Li

**Affiliations:** 1 School of Information Technology and Mathematical Sciences, University of South Australia, Adelaide, South Australia, Australia; 2 Faculty of Engineering, Dali University, Dali, China; 3 Department of Computer Science, Zhejiang Normal University, China; University of Erlangen-Nuremberg, GERMANY

## Abstract

microRNAs (miRNAs) are important gene regulators at post-transcriptional level, and inferring miRNA-mRNA regulatory relationships is a crucial problem. Consequently, several computational methods of predicting miRNA targets have been proposed using expression data with or without sequence based miRNA target information. A typical procedure for applying and evaluating such a method is i) collecting matched miRNA and mRNA expression profiles in a specific condition, e.g. a cancer dataset from The Cancer Genome Atlas (TCGA), ii) applying the new computational method to the selected dataset, iii) validating the predictions against knowledge from literature and third-party databases, and comparing the performance of the method with some existing methods. This procedure is time consuming given the time elapsed when collecting and processing data, repeating the work from existing methods, searching for knowledge from literature and third-party databases to validate the results, and comparing the results from different methods. The time consuming procedure prevents researchers from quickly testing new computational models, analysing new datasets, and selecting suitable methods for assisting with the experiment design. Here, we present an R package, miRLAB, for automating the procedure of inferring and validating miRNA-mRNA regulatory relationships. The package provides a complete set of pipelines for testing new methods and analysing new datasets. miRLAB includes a pipeline to obtain matched miRNA and mRNA expression datasets directly from TCGA, 12 benchmark computational methods for inferring miRNA-mRNA regulatory relationships, the functions for validating the predictions using experimentally validated miRNA target data and miRNA perturbation data, and the tools for comparing the results from different computational methods.

## Introduction

miRNAs are important gene regulators controlling a wide range of biological processes and are involved in several types of cancers (see [1] for a review). Thus, exploring miRNA functions is important for diagnostics and therapeutics. However, there are still no feasible experimental techniques to discover miRNA regulatory mechanisms. Computational methods are proved to be an effective approach to exploring miRNA functions by predicting miRNA-mRNA regulatory relationships. These prediction methods help reduce the number of experiments that must be conducted and assist with the design of the experiments.

There is a large amount of data and tools for predicting miRNA targets. Sequence based prediction methods, which use the principal of sequence complementary and/or structural stability of the putative duplex, provide genome wide predictions of miRNA targets, but the results may contain a high rate of false discoveries [2]. Meanwhile, gene expression based methods are normally proposed to infer the miRNA-mRNA regulatory relationships in a specific condition, which can be classified as correlation based analysis [3, 4], regression models [5, 6], Bayesian inference [7], and causal inference [8–10]. Each of the methods has its own merits and different methods may discover complementary results [11]. There are also methods which integrate both sequence based target information and gene expression data for identifying miRNA targets [7, 12–14]. The computational methods help broaden our understanding of miRNA functions and generate hypotheses for wet lab experiments.

However, there is still a lack of tools which help researchers to quickly evaluate new computational methods, analyse new datasets, and select suitable methods for assisting with the design of experiments. It is time consuming for both bioinformaticians and biologists to test new ideas and explore miRNA functions. Bioinformaticians may need to go through a long process of searching and pre-processing the matched miRNA and mRNA datasets, repeating the computational methods and applying them to the input datasets, evaluating the predictions, and comparing the performance of their proposed method with other benchmark methods. Biologists may find it difficult to quickly explore the regulatory relationships in their new data using some computational methods, or select a suitable model for generating hypotheses for experiment design.

To fill this gap, here we present an R package, miRLAB, to provide a comprehensive facility for exploring and experimenting with miRNA-mRNA regulatory relationships. miRLAB provides a dry or computational laboratory on a single computer where one can load or retrieve datasets, test or apply new or existing computational methods, and validate predicted results, all in an automated manner.

miRLAB contains a set of commonly used miRNA and mRNA expression datasets and a pipeline to retrieve datasets from TCGA, and the scripts for pre-processing gene expression data such as differently expressed gene analysis. A number of easy-to-use benchmark computational methods for inferring miRNA-mRNA regulatory relationships are embedded in miRLAB (normally just one line of code for function call for each of the computational methods). miRLAB includes functions for validating the predictions using experimentally validated miRNA target data and miRNA perturbation data, and functions for enrichment analysis of miRNA target genes.

Users can use the package in different scenarios, including using the processed data included in miRLAB, TCGA data, and/or user provided datasets for testing a new computational method developed by a user, by comparing the prediction results with those predicted by the embedded benchmark methods; investigating the regulatory relationships in cancers by applying a benchmark method to cancer datasets from TCGA or a dataset provided by a user; or comparing the performance of different methods on a new dataset given by user in order to select a suitable prediction method for assisting with the experiment design.

The miRLAB package is implemented in R and is freely available on Bioconductor at http://bioconductor.org/packages/miRLAB/. The package will be updated with new datasets, benchmark computational methods, and experimentally confirmed data when they are available. We hope that miRLAB will speed up miRNA research activities.

## Materials and Methods

### Workflow of miRLAB

**Fig 1:**
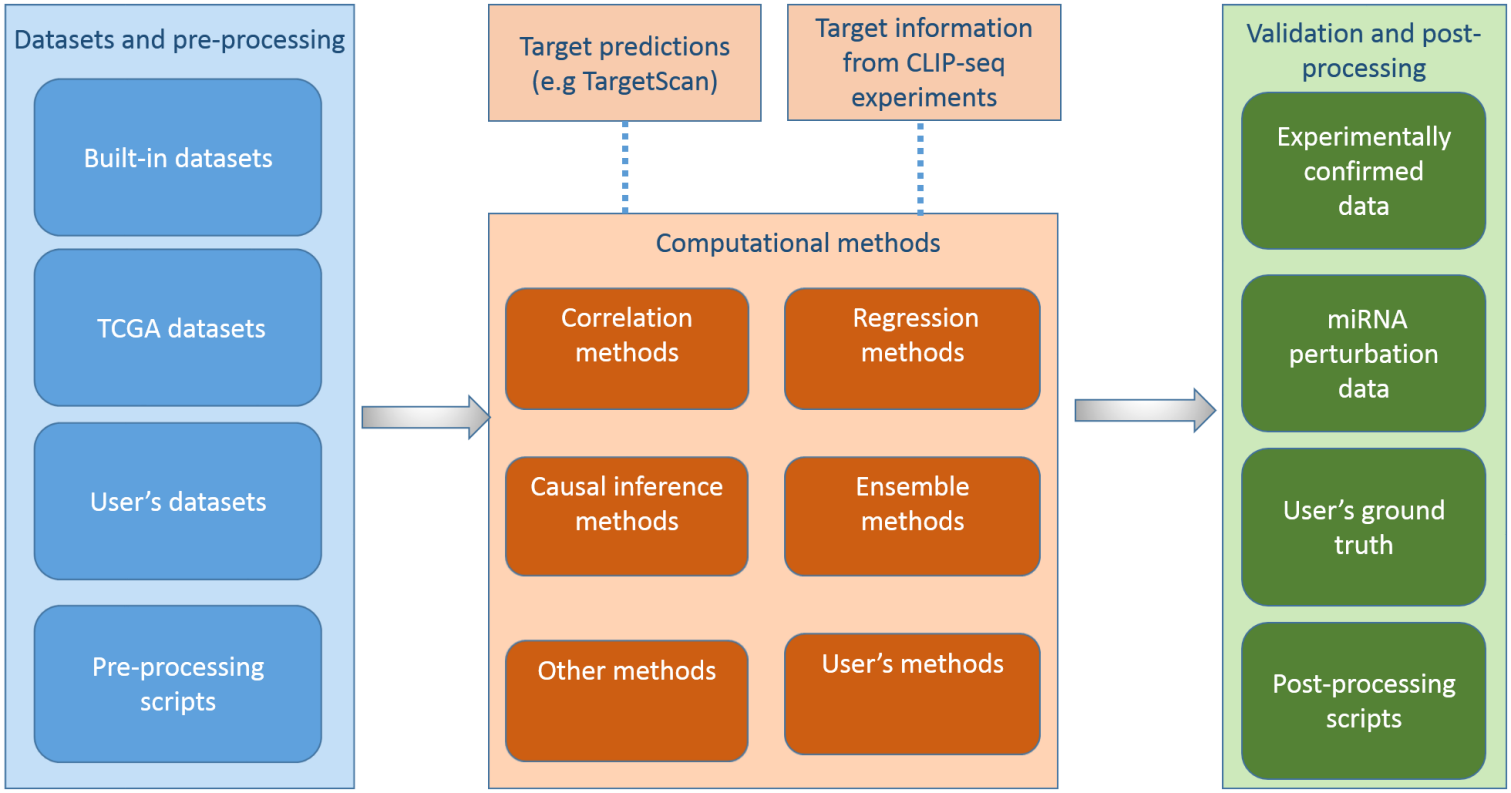
The workflow of miRLAB package. The workflow mainly includes three components: Datasets and pre-processing, Computaional methods for exploration, and Validation and post-processing. It is optional to integrate miRNA target predictions and/or miRNA target binding information from CLIP-seq experiments to the computational models. Users can provide their own datasets/methods in each step of the workflow.

As illustrated in Fig 1, miRLAB package provides a pipeline consisting of three components to identify and validate miRNA-mRNA regulatory relationships. In the following, we describe the workflow of miRLAB package in detail.

### Datasets and pre-processing

We provide 3 processed gene expression datasets that can be downloaded from the package website at: http://sourceforge.net/projects/mirlab/files/?source=navbar, including the EMT (Epithelial to Mesenchymal Transition) [15], MCC (Multi-Class Cancer) [16, 17], and PAN (Pan-Cancer in TCGA) [18] datasets. We use the limma package [19] of Bioconductor to identify differentially expressed miRNAs and mRNAs. As a result of differential expression (DE) analysis, 35 miRNAs and 1154 mRNAs in the EMT dataset (adjusted p-value < 0.05), and 108 miRNAs and 1860 mRNAs in the MCC dataset (adjusted *p*-value < 0.05) are identified to be differentially expressed. Meanwhile, 103 miRNAs (adjusted *p*-value < 10E-03) and 1553 mRNAs (adjusted *p*-value < 10E-06) in the PAN cancer dataset are identified to be differentially expressed. The original expression dataset without the DE analysis are also available at the package website. Using the *DiffExpAnalysis* function in the package, users will be able to set their own *p*-value thresholds for each dataset in the DE analysis.

We also provide the pipeline for downloading matched miRNA and mRNA expression profiles for cancer datasets from TCGA based on TCGA Assembler [20]. Users can use abbreviated cancer names as the input of the *getData* function of miRLAB to obtain matched miRNA and mRNA expression profiles in specific cancer.

In addition, users can input their own datasets. It is noted that the input datasets should include matched samples of miRNA and mRNA expression data.

miRLAB provides several utilities for pre-processing input data, including data standardisation (the *Standardise* function), missing value imputation and data normalisation (the *ImputeNormData* function), and DE analysis (the *DiffExpAnalysis* function). Moreover, we have a function, *convert*, to convert between miRBase versions (currently versions 16-21) for a list of miRNAs. This function helps solve the problem of having multiple datasets with different miRBase versions of miRNAs. We use miRBase version 17 for all miRNAs in our package, but users can change the miRBase version of the miRNAs in the output results to any other miRBase version using the *convert* function. We will also update the package when new versions of miRBase become available (and stable).

### Computational methods

In miRLAB, we provide a number of commonly used computational methods for miRNA target prediction. We have re-implemented the methods so that they are easy to use with a simple function call (just one line of code for calling most of the methods). We discuss the details of each of the implemented methods in the following. We will also describe how users can load an external method and run it in miRLAB, as well as how sequence based target information can be utilised with the embedded methods and user provided methods.

#### Correlation methods

Pearson’s correlation coefficient [21] is the commonly used measure for the strength of the association between a pair of variables. For the miRNA target prediction problem, the correlations in the expression levels between pairs of miRNA and mRNA are calculated and the miRNA-mRNA pairs are ranked based on the correlation coefficient values. As miRNAs mainly down-regulate mRNAs, negative correlations are ranked at the top.

Apart from Pearson’s correlation coefficient, in miRLAB we have implemented the other popular correlation methods, including Spearman [22], Kendall [23], Distance correlation (Dcov) [24], Hoeffding’s D measure (Hoeffding) [25], and Randomised Dependence Coefficient (RDC) [26].

As correlation based methods are designed to capture only linear associations, and their usefulness may be greatly reduced when the associations are non-linear [27], we also include the Mutual Information (MI) method [28] in the package to deal with non-linear relationship discovery.

#### Regression methods

Lasso [29] and Elastic-net [30] are popular high-dimensional regression techniques which can be used to infer the association between variables. In our problem, each mRNA is presented as a linear expression of all miRNAs, and the coefficient of a miRNA in the regression model is used as the association strength between the miRNA and the mRNA. For each mRNA, we can perform Lasso or Elastic-net regression on all the miRNAs, using the R package *glmnet* [31]. Similar to the correlation methods, the negative miRNA-mRNA effects are ranked at the top of the list to favour down-regulation.

#### Causal inference methods

Maathuis et al. [32, 33] proposed a causal inference method, called Intervention calculus when the DAG (Directed Acyclic Graph) is Absent (IDA), which estimates the causal effect that a variable has on the other. The estimated causal effects simulate the effects of randomised controlled experiments. Recently, Le et al. [8] applied this method to gene expression datasets to infer the causal (regulatory) relationships between miRNAs and mRNAs. The discovered miRNA-mRNA causal regulatory relationships were found to have a large portion of overlap with the results of the follow-up gene knockdown experiments. Therefore, we include this method in miRLAB as one of the benchmark methods.

#### Other methods

Z-score [34] is a network inference method to estimate the effects of gene knockout experiment. When we knockout a miRNA, Z-score describes the normalised deviation of the expression level of each gene from the average expression of all genes. However, in our study, we use observational data, i.e. gene expression data, as the input dataset. Therefore, the Z-score method is modified where we assume that the minimum expression value of the miRNA in a profile indicates the knockout experiment. In other words, we firstly locate the sample that contains the minimum value of the miRNA, and then we use the expression value of the mRNA in the sample to calculate Z-score.

ProMISe [12] assumes that there is a competition between miRNAs to be attracted by a mRNA and between mRNAs to attract a miRNA for interactions. The method estimates the probability of a mRNA to be a target of the miRNA taking the competition between mRNAs and the competition between miRNAs into account. The method is implemented in the R package *Roleswitch* from Bioconductor.

#### Ensemble methods

We also provide the scripts for creating ensemble methods from different individual methods. The ensemble methods use the Borda count election method [35] to integrate the results from different individual methods. The aim of designing an ensemble method is to discover complement results using different individual methods [36]. In our implementation, we provide two slightly different versions of ensemble generation. The first version is faithful to the Borda rank idea, which outputs the average rank of all rankings from individual methods. In the second version, we do not use the whole ranking of each method to calculate the average, but only use the top *k* targets in each ranking. The second version is designed to appreciate the genes that are predicted within the top *k* lists across methods. Genes outside the top *k* list of a method is assigned the maximal ranking for that method, i.e. the maximum number of genes in the dataset. Although there is no theoretical background behind choosing the optimal *k*, the second version provides a tool for users to customise their experiments. For example, ones may want to find the targets of a miRNA predicted by the ensemble of three different methods, but they believe only the top 100 targets predicted by a method are of their interest, then in this case, the user can set *k* = 100.

#### Incorporating sequence based miRNA target information and gene expression data

While gene expression data provides information of gene activities in a specific condition, sequence based target information indicates the actual binding activities between miRNAs and their target genes. In miRLAB, we provide an option to incorporate the target information from different sequence based prediction programs, e.g. TargetScan v6.2 [37], TargetScan v7.0 [38], DIANA-microT-CDS [39], or CLIP experiments, such as HITS-CLIP (also known as CLIP-Seq) [40], PAR-CLIP [41] and iCLIP [42] into each of the computational methods. We provide those databases in the package website. The results obtained by incorporating the target information will be the interactions that are not only identified by the computational methods using expression data but also predicted based on sequence information.

### Validation and post-processing

It is difficult to validate computational results, as the number of experimentally confirmed targets of miRNAs is still limited and there is no complete ground truth for evaluating and comparing different computational methods [11]. In miRLAB, we provide two kinds of ground truth to validate the predictions of a computational method: experimentally confirmed miRNA target data and miRNA perturbation data.

We firstly use the union of four popular experimentally confirmed target databases, TarBase [43], miRecords [44], miRWalk [45], and miRTarBase [46] to validate the predictions of the methods for all miRNAs. Respectively for Tarbase, miRecords, miRWalk, and miRTarBase, we have 20095 interactions with 228 miRNAs, 21590 interactions with 195 miRNAs, 1710 interactions with 226 miRNAs, and 37372 interactions with 576 miRNAs. After removing the duplicates, we have in total 62858 unique interactions for validations. Some of the interactions in miRTarBase and miRecords are validated using negative correlations in expression data only. We refer these interactions as weakly validated interactions, and provide another version of ground truth in which weakly validated interactions are removed. Both versions of the ground truth (with and without weakly validated interactions being removed) are available at the package website.

Secondly, we use the curated perturbation experiments from 177 tissues from [47] to validate the computational predictions. Perturbation experiments measure the gene expression levels in two groups, control group (without the miRNA of interest) and transfected group (with the miRNA of interest). The change in expression level of a gene in the two groups represents the effect of the miRNA on the gene. We use the log2 fold-change (LFC) 1.0 as the threshold for cutting off the genes without significant changes. The genes with |*LFC*|>1 will be considered as the targets of the miRNA in the perturbation experiment.

To understand the underlying biological processes and pathways of miRNA targets, we provide the pipeline for functional and pathway enrichment analysis, including two functions (*GOBPenrichment* and *KEGGenrichment*) to conduct GO (Gene Ontology) [48] and KEGG (Kyoto Encyclopedia of Genes and Genomes) [49] enrichment analysis. Users only need to provide a list of gene symbols of interest and a p-value cutoff to get significant GO biological processes and KEGG pathways.

## Results

The miRLAB package is designed to assist with exploring miRNA-mRNA regulatory relationships. The package is implemented in R and is available as a Bioconductor package at http://bioconductor.org/packages/miRLAB/. In the following, we present some typical scenarios of using the package with different purposes. All relevant datasets are available at http://sourceforge.net/projects/mirlab/files/?source=navbar. In this section, we demonstrate the usages of miRLAB in different scenarios.

### Scenario 1: Using a built-in dataset for testing a new method

In this scenario, we suppose that a developer has created a new miRNA target prediction method and would like to quickly test its performance on a matched miRNA and mRNA gene expression dataset. We use the EMT dataset for demonstration in this scenario. The new method should return a matrix of prediction scores. Each cell *c*_*ij*_ of the matrix represents the score of the prediction that the *i* ^*th*^ mRNA is a target of the *j* ^*th*^ miRNA. As an example, we assume further that the new method is an ensemble method which combines the results of the Pearson correlation coefficient method and IDA. As illustrated below the whole procedure of creating and testing the new method includes only some lines of codes.

**Fig 2:**
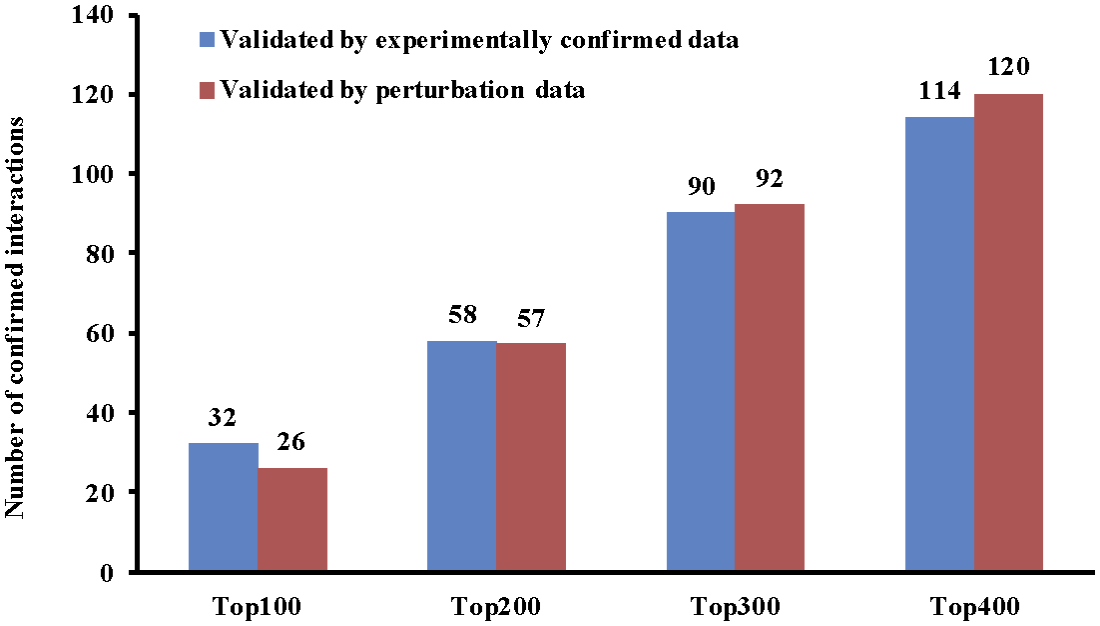
The number of confirmed miRNA-mRNA interactions by experimentally confirmed data and perturbation data. The top 100, 200, 300, and 400 interactions for each miRNA are selected for the validation in the EMT dataset.

If we validate the results predicted by the new method for the top 100, 200, 300 and 400 targets of each miRNA, we will come up with the total number of confirmed interactions shown in Fig 2.

#Assume that the EMT35.csv has been downloaded from

#http://sourceforge.net/projects/mirlab/files/?source=navbar

#and placed in the working directory

library(miRLAB)

PearsonEMT=*Pearson*(“EMT35.csv”, cause=1:35, effect=36:1189)

IDAEMT=*IDA*(“EMT35.csv”, cause=1:35, effect=36:1189, “stable”, 0.01)

NewMethod=*Borda*(list(PearsonEMT, IDAEMT))

#Validate the top 100 targets of each miRNA

#Assume that the groundtruth.csv is placed in the current directory

Result100=*ValidateAll*(NewMethod, topk=100, “groundtruth.csv”,1.0)

### Scenario 2: Comparing results of different methods on a new dataset (user’s dataset)

**Fig 3:**
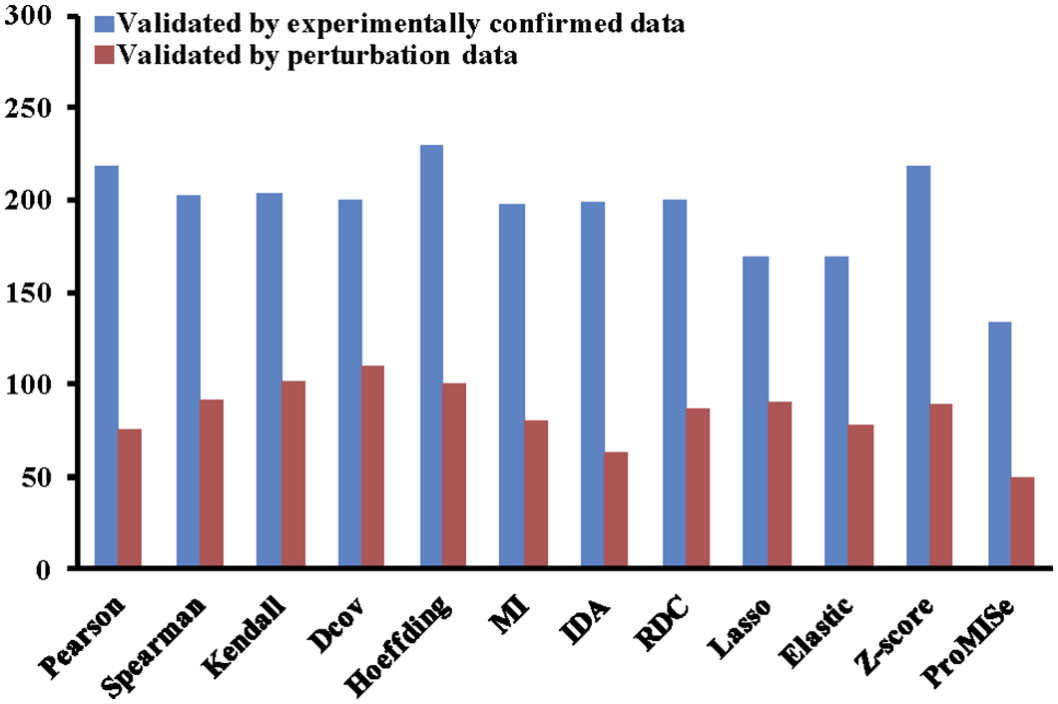
Comparison of the 12 built-in miRNA target prediction methods. The experimentally confirmed data and perturbation data are used for the validation in the MCC dataset. The top 100 targets of each miRNA are extracted for validation.

In this scenario, we assume that we have a new dataset and would like to apply different computational methods to the dataset and validate the results against current literature knowledge. As an example, we use the MCC dataset as the new dataset. In the following, we show the R code to compare the results of the 12 built-in methods for the MCC dataset. For each miRNA, we extract the top 100, 200, 300, and 400 target genes ranked by each method for validation. We only keep the miRNAs that have at least one confirmed target predicted by all the methods. We calculate the ranking score of each method for the MCC dataset by summing up its ranking scores for all miRNAs. The higher the ranking score of a method, the better the method is. As shown in Fig 3, when using experimentally confirmed data as ground truth, Pearson correlation coefficient performs the best. If the ground truth is perturbation data, Hoeffding outperforms the other methods.

#Assume that “MCC.csv” plays the role of a user’s dataset

#and has been placed in the working directory

library(miRLAB)

cause=1:108   #in the MCC dataset, the first 108 columns are miRNAs

effect=109:1968

pearson=*Pearson*(“MCC.csv”, cause, effect)

spearman=*Spearman*(“MCC.csv”, cause, effect)

kendall=*Kendall*(“MCC.csv”, cause, effect)

dcov=*Dcov*(“MCC.csv”, cause, effect)

hoeffding=*Hoeffding*(“MCC.csv”, cause, effect)

mi=*MI*(“MCC.csv”, cause, effect)

ida=*IDA*(“MCC.csv”, cause, effect, “stable”, 0.01)

rdc=*RDC*(“MCC.csv”, cause, effect)

lasso=*Lasso*(“MCC.csv”, cause, effect)

elastic=*Elastic*(“MCC.csv”, cause, effect)

zscore=*Zscore*(“MCC.csv”, cause, effect)

promise=*ProMISe*(“MCC.csv”, cause, effect)

all=list(pearson, spearman, kendall, dcov, hoeffding, mi, ida, rdc, lasso, elastic, zscore, promise)

allresultsTop100=*experiment*(all, topk=100, “groundtruth.csv”,1.0)

compareTop100=*filterAndCompare*(allresultsTop100, 1)

### Scenario 3: Running a benchmark method for a TCGA cancer dataset

**Fig 4:**
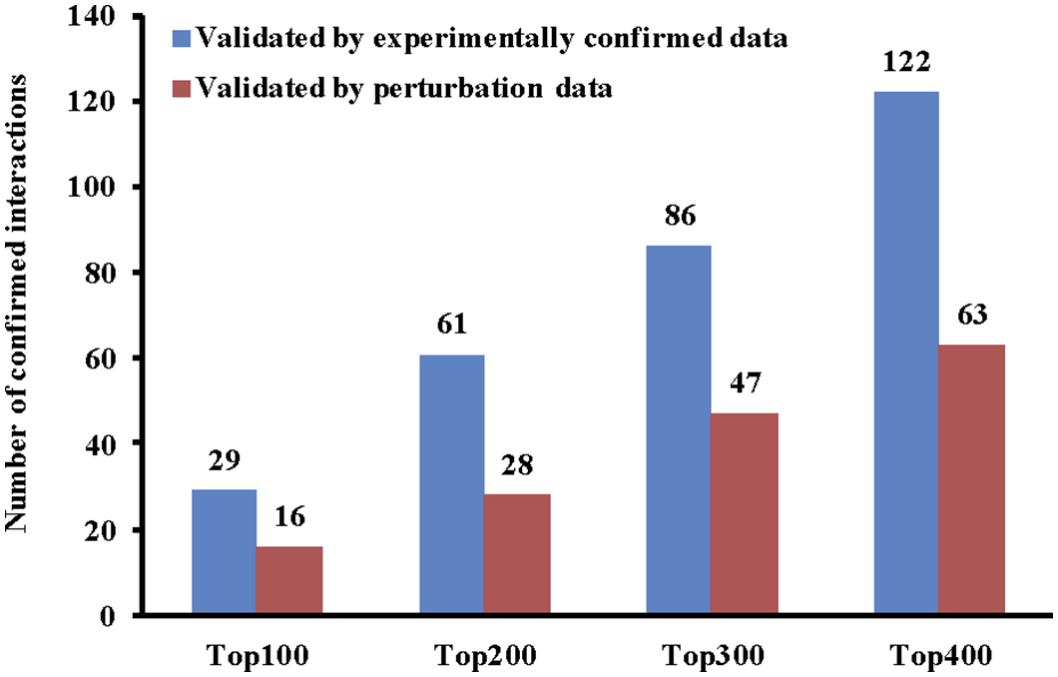
The number of confirmed miRNA-mRNA interactions by experimentally confirmed data and perturbation data. The top 100, 200, 300, and 400 interactions for each miRNA are selected for the validation in the PRAD dataset from TCGA.

It is very useful to utilise the wealth amount of data in TCGA for exploring miRNA functions in cancer. In this scenario, we assume that we would like to apply a causal inference method (IDA) for predicting miRNA targets in the Prostate adenocarcinoma (PRAD) dataset. We will firstly use the built-in *getData* function to get the matched samples of miRNA and mRNA expression profiles from TCGA directly. We can then conduct the DE analysis for the downloaded PRAD dataset using the function *DiffExpAnalysis*. After pre-processing the dataset, we use IDA to infer miRNA-mRNA interactions and validate them by using two types of ground truth (experimentally confirmed data and miRNA perturbation data). The R code for the whole process is shown in the following. If we respectively validate the top 100, 200, 300, 400 targets of each miRNA and summarise the results, we will have the total number of confirmed interactions as shown in Fig 4.

library(miRLAB)

RawPRAD=*getData*(“PRAD”)

write.csv(RawPRAD,“RawPRAD.csv”)

#Diffrentially expressed analysis.

#Preparing csv files for miRNA and mRNA in each condition,

#e.g. tumor vs normal.

PRAD=*DiffExpAnalysis*(“miRNA_Tumor.csv”,“miRNA_Normal.csv”, “mRNA_Tumor.csv”,“mRNA_Normal.csv”,50,1000)

write.csv(PRAD,“PRAD-50.csv”, row.names=FALSE)

datacsv=“PRAD-50.csv”

idaPRAD=*IDA*(datacsv, cause=1:50, effect=51:1050, “stable”, 0.01)

ResultsPRAD100=*ValidateAll*(idaPRAD, topk=100, “Groundtruth”,1.0)

### Scenario 4: Using target information together with a built-in method

**Fig 5:**
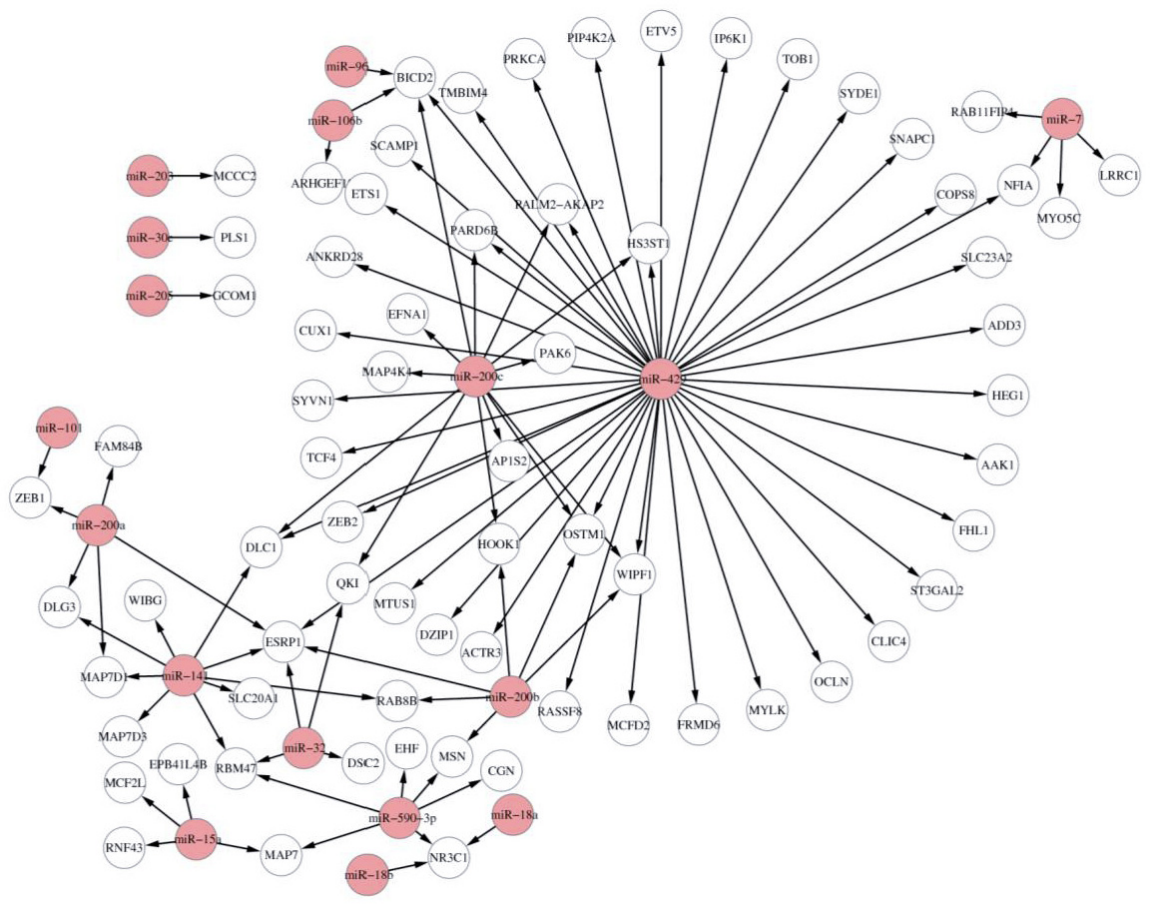
Visualisation of top 100 miRNA-mRNA interactions for all miRNAs in the EMT dataset. TargetScan is used as the putative target information. The pink nodes are miRNAs, and the white nodes are mRNAs.

In this scenario, we incorporate target binding information and gene expression data to infer the miRNA-mRNA relationships. Suppose that we use TargetScan v6.2 as target information together with IDA as the computational method to infer miRNA-mRNA interactions. We use the built-in EMT dataset for demonstration in this scenario. In order to understand the potential biological processes and pathways of the top miRNA-mRNA interactions, we also make GO and KEGG enrichment analysis of miRNA targets using the *GOBPenrichment* and *KEGGenrichment* functions, respectively. The code for running this procedure is as follows and the top 100 miRNA-mRNA interactions for all miRNAs are shown in Fig 5. The results of significant GO and KEGG terms (adjusted *p*-value <0.05) are found in S1 File.

library(“miRLAB”)

#Assume that the “EMT.csv” dataset and the target information file,

#“Targetscan.csv”, have been placed in the working directory.

idaTargetscan =*IDA*(datacsv, cause=1:35, effect=36:1189, “stable”, 0.01, targetbinding=“TargetScan.csv”)

idaTop100=*Extopk*(idaTargetscan,topk=100)

idaGO=*GOBPenrichment*(unique(idaTop100[,2]),0.05)

idaKEGG=*KEGGenrichment*(unique(idaTop100[,2]),0.05)

### Scenario 5: Comparing results predicted from external methods

**Fig 6:**
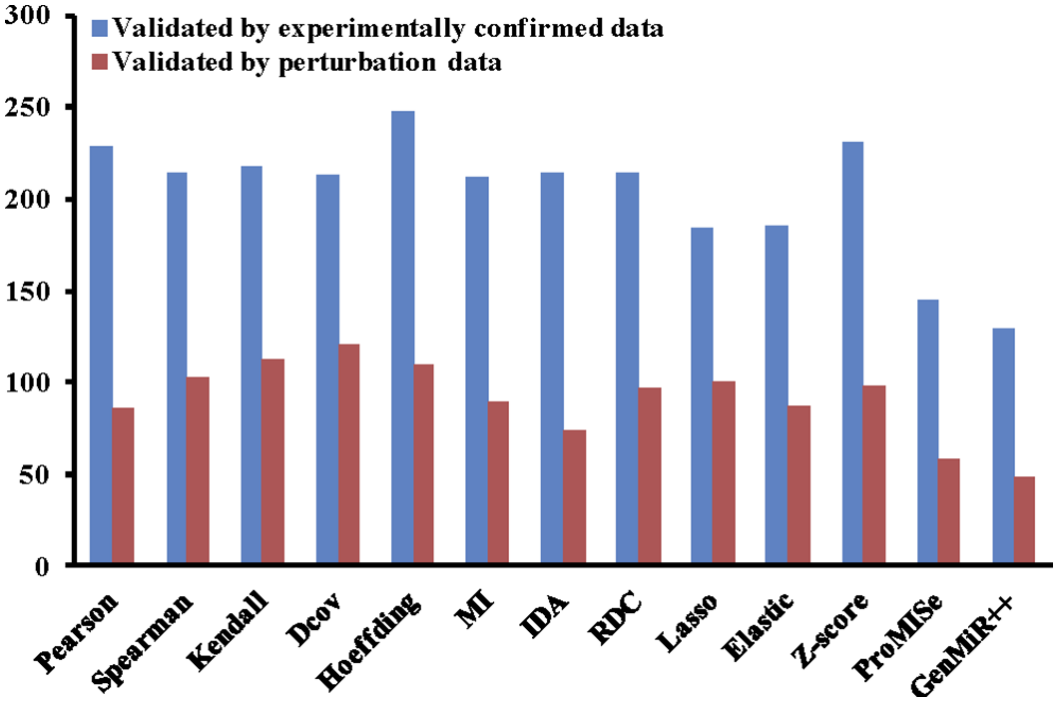
Comparison of the 12 built-in miRNA target prediction methods and the external method GenMiR++. The experimentally confirmed data and perturbation data are used for the validation of the results the MCC dataset. The top 100 targets of each miRNA are extracted for validation.

There are other miRNA target prediction methods that are either implemented in R or in other programming languages. It is possible to validate the predicted results from those methods using miRLAB, and comparing the performance of those methods with the miRLAB built-in methods. In this scenario, we demonstrate how to validate the results generated by GenMiR++ [7], which was implemented in Matlab. The output result of GenMiR++ for the MCC dataset is available on the package website at: http://sourceforge.net/projects/mirlab/files/?source=navbar. We can also compare the performance of the external method with other built-in methods in miRLAB. Fig 6 shows the comparison results of GenMiR++ and the 12 methods in miRLAB for the MCC dataset using the ranking scores as in Scenario 2.

library(“miRLAB”)

#Assume that the “MCC.csv” dataset and the GenMiR++ output file,

#“gmMCC.csv”, have been placed in the working directory.

cause=1:108   #in the MCC dataset, the first 108 columns are miRNAs

effect=109:1968

#Read results predicted by GenMiR++

genmir= *ReadExtResult*(“MCC.csv”, cause, effect, “gmMCC.csv”)

#Validate the top 100 targets of each miRNA

#Assume that the groundtruth.csv is placed in the current directory

Result100=*ValidateAll*(genmir, topk=100,“groundtruth.csv”,1.0)

## Discussions

In this paper, we provide an R environment to explore miRNA functions. The 12 built-in methods can be applied to expression data with or without target information. As we do not have the complete ground truth for evaluating the models, it is impossible to conclude which method is better than the other. Using the current knowledge on the experimentally confirmed miRNA targets, our previous experimental results show that there is no superior method that outperforms all other methods in the three datasets [36]. Moreover, different computational methods may predict different sets of miRNA targets [11], suggesting that each method has their own merits. In the following, we briefly discuss the characteristics of different groups of computational methods which may serve as a guide for selecting the methods for a particular dataset.

Correlation and mutual information methods are suitable for high dimensional datasets. Since these groups of methods only consider the pairwise relationships between a miRNA and a mRNA, the methods are good candidates for datasets with large number of features (miRNAs and mRNAs). The assumption on the relationships between miRNAs and mRNAs will decide which method in this category should be used. For example, if we assume linear relationships between miRNAs and mRNAs, Pearson correlation coefficient would be suitable. Meanwhile, for non-linear relationships, a mutual information method should be chosen.

For datasets with small number of miRNAs, regression methods would be great candidates. In the regression model, a mRNA is represented as a function of all miRNAs in the dataset. Therefore, with relatively small number of samples in real world datasets, the regression methods would achieve better results when we do not have too many miRNAs in a dataset.

The causal inference methods have been demonstrated to be effective in removing spurious relationships. However, the complexity of the causal inference is high and therefore it would be suitable for datasets with relatively small number of features (miRNAs and mRNAs). Alternatively, these causal inference methods would require high performance computers to run with large datasets.

In the general case, users may want to use an ensemble method which integrates the predictions from different methods. The ensemble approach has been proved theoretically and experimentally to outperform individual methods across different datasets [35], especially, when integrating methods from different approaches. In the package we provide a function to generate an ensemble method by combining different built-in computational methods. Moreover, users can also read results from any external methods into R using the *ReadExtResult* function as demonstrated in Scenario 5, and then the ensemble method can be formed by combining the external methods or combining external methods with the built-in methods. This would help users create new ensemble methods when novel computational methods become available in the future.

## Conclusion

In recent years, miRNAs have been found to play pivotal roles at the post-transcriptional level. By fine-tuning the expression levels of target genes, miRNAs play important roles in many biological processes and different diseases, including cancer. Thus, exploring miRNA-mRNA regulatory relationships is vital in understanding biological processes and may provide insights into the regulatory mechanism of miRNAs in the pathophysiology of diseases.

To date, several computational methods have been proposed to infer miRNA-mRNA regulatory relationships using expression data with or without miRNA target information. Each of the methods has its own merits and no single method always performs the best in all datasets. There is still a lack of tools for evaluating computational methods and exploring miRNA functions in a new dataset, validating miRNA prediction results, and selecting suitable methods for assisting experiment design.

To address the problem, we propose the idea of creating a comprehensive dry lab environment on a single computer. Following the idea, we have developed an R package called miRLAB, to provide such a computational lab for exploring and experimenting with miRNA-mRNA regulatory relationships. miRLAB includes three components: Datasets and pre-processing, Computational methods, and Validation and post-processing. In this paper, we have used five different scenarios to show how to use the package. Details of all functions are listed in the user manual and users may design their own workflow using those easy-to-use functions.

For the miRLAB package, we have implemented the commonly used computational methods for miRNA target prediction, but newly developed methods will be integrated into the package constantly. Furthermore, we currently use two types of ground truths, i.e. experimentally validated miRNA target data and miRNA perturbation data, to validate miRNA prediction results. However, the data in those ground truths are far from complete, and we will continuously update the data in our package in line with the updates of the original databases. Moreover, miRNAs tend to regulate mRNAs in specific conditions, i.e. the interactions occur under some specific biological conditions but not under the other conditions. However, current experimentally validated databases do not provide a systematic way to get the biological conditions of the interactions. In the future, we will classify the confirmed interactions into specific cell lines, tissues, and other biological conditions. We hope that the classified confirmed interactions would provide more insights into miRNA functions in a particular dataset. Finally, the miRLAB package only focuses on inferring miRNA-mRNA regulatory relationships. We will extend the scope of the package to cover other gene regulators such as transcription factors and long non-coding RNAs in the future.

## Supporting Information

S1 File Significant GO terms and KEGG pathways generated in Scenarios 4. (XLSX)
